# Interaction of Mannose-Binding Lectin With Lipopolysaccharide Outer Core Region and Its Biological Consequences

**DOI:** 10.3389/fimmu.2018.01498

**Published:** 2018-06-29

**Authors:** Aleksandra Man-Kupisinska, Anna S. Swierzko, Anna Maciejewska, Monika Hoc, Antoni Rozalski, Malgorzata Siwinska, Czeslaw Lugowski, Maciej Cedzynski, Jolanta Lukasiewicz

**Affiliations:** ^1^Laboratory of Microbial Immunochemistry and Vaccines, Department of Immunochemistry, Ludwik Hirszfeld Institute of Immunology and Experimental Therapy, Polish Academy of Sciences, Wroclaw, Poland; ^2^Laboratory of Immunobiology of Infections, Institute of Medical Biology, Polish Academy of Sciences, Lodz, Poland; ^3^Department of Biology of Bacteria, Faculty of Biology and Environmental Protection, Institute of Microbiology, Biotechnology and Immunology, University of Lodz, Lodz, Poland; ^4^Laboratory of General Microbiology, Faculty of Biology and Environmental Protection, Institute of Microbiology, Biotechnology and Immunology, University of Lodz, Lodz, Poland

**Keywords:** lipopolysaccharide, endotoxin, anaphylactoid shock, complement, mannose-binding lectin, *Hafnia*

## Abstract

Lipopolysaccharide (LPS, endotoxin), the main surface antigen and virulence factor of Gram-negative bacteria, is composed of lipid A, core oligosaccharide, and O-specific polysaccharide (O-PS) regions. Each LPS region is capable of complement activation. We have demonstrated that LPS of *Hafnia alvei*, an opportunistic human pathogen, reacts strongly with human and murine mannose-binding lectins (MBLs). Moreover, MBL–LPS interactions were detected for the majority of other Gram-negative species investigated. *H. alvei* was used as a model pathogen to investigate the biological consequences of these interactions. The core oligosaccharide region of *H. alvei* LPS was identified as the main target for human and murine MBL, especially l-*glycero*-d-*manno*-heptose (Hep) and *N*-acetyl-d-glucosamine (GlcNAc) residues within the outer core region. MBL-binding motifs of LPS are accessible to MBL on the surface of bacterial cells and LPS aggregates. Generally, the accessibility of outer core structures for interaction with MBL is highest during the lag phase of bacterial growth. The LPS core oligosaccharide–MBL interactions led to complement activation and also induced an anaphylactoid shock in mice. Unlike *Klebsiella pneumoniae* O3 LPS, robust lectin pathway activation of *H. alvei* LPS *in vivo* was mainly the result of outer core recognition by MBL; involvement of the O-PS is not necessary for anaphylactoid shock induction. Our results contribute to a better understanding of MBL–LPS interaction and may support development of therapeutic strategies against sepsis based on complement inhibition.

## Introduction

Mannose-binding lectin (MBL) is one of several pattern recognition molecules forming complexes with MBL-associated serine proteases (MASP) able to activate complement *via* the lectin pathway (LP). That process contributes to clearance of infection, but when excessive may be detrimental to the host (1).

Humans synthesize one type of MBL (hMBL), whereas mice (like the majority of mammals) synthesize two forms, MBL-A and -C, differing slightly in their specificity, serum concentration, activity, and local expression (2–5). Generally, hMBL recognizes carbohydrate patterns present on pathogens that are rich in d-mannose (d-Man), *N*-acetyl-d-glucosamine (d-GlcNAc), *N*-acetyl-d-mannosamine (d-ManNAc), or l-fucose (l-Fuc).

**Figure 1:**
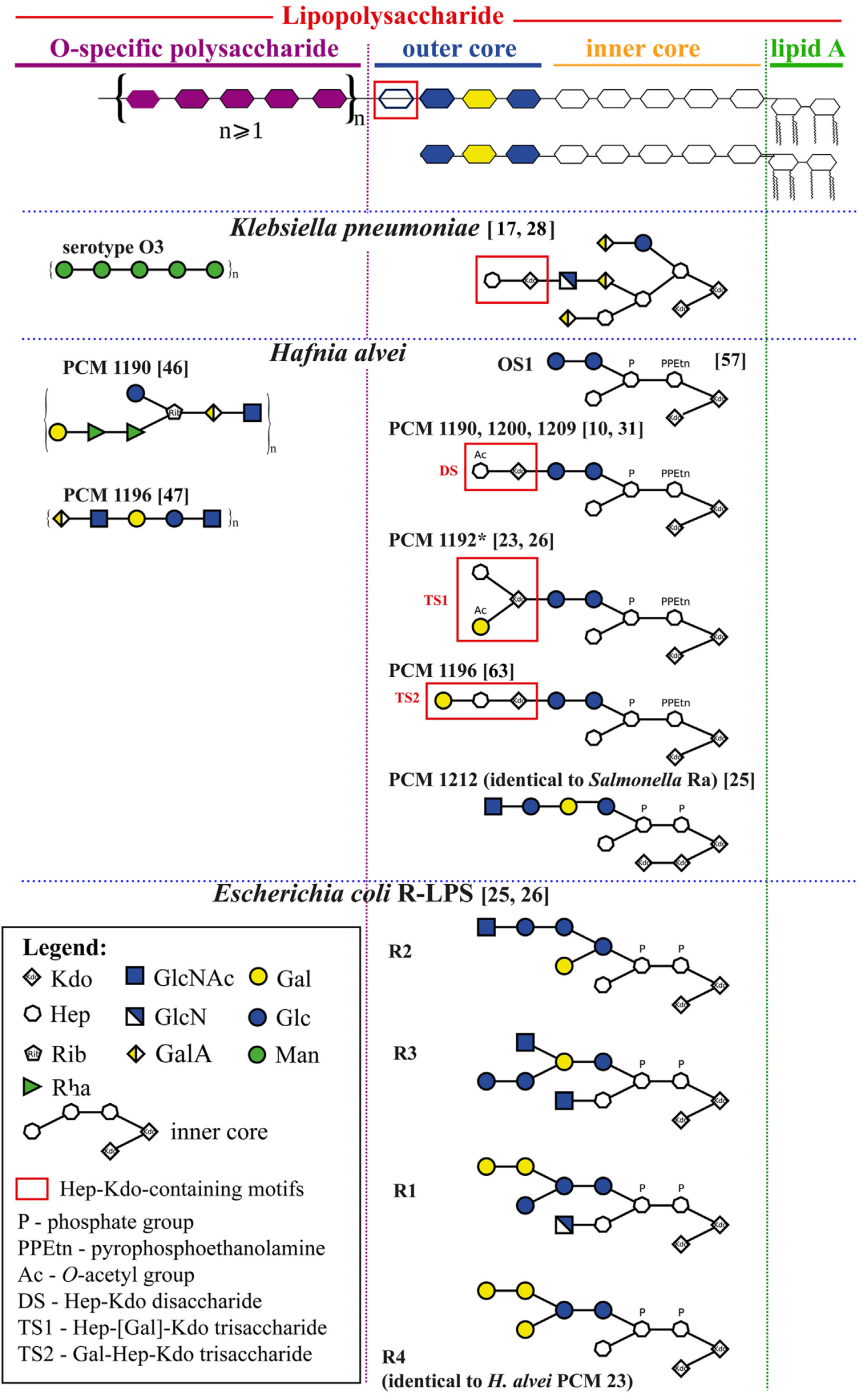
Schematic structures of selected *Klebsiella pneumoniae, Hafnia alvei*, and *E. coli*, and *Salmonella* spp. lipopolysaccharides (LPSs) relevant for an interpretation of human mannose-binding lectin–LPS interactions. All structures are grouped according to the schematic diagram of an LPS molecule (upper panel) built of lipid A, core oligosaccharide (inner and outer), and O-specific polysaccharide consisting of a varying number of oligosaccharide repeating units. Information about linkages and isomers was hidden to simplify an interpretation of structures and may be found in details in references (numbers in brackets).

Lipopolysaccharide (LPS, endotoxin), the main surface antigen of Gram-negative bacteria, may be a ligand of MBL. LPS is composed of lipid A linked to a core oligosaccharide (OS) consisting of inner and outer regions that is further substituted with O-specific polysaccharide (O-PS) comprising oligosaccharide repeating units. O-PS is a very variable region that determines O-serotype, whereas core OS and lipid A are characterized by moderate structural variability. Smooth bacterial strains synthesize highly heterogeneous LPS being the mixture of S-LPS built of all three regions and short R-LPS (devoid of the O-PS) (Figure 1). Rough bacteria synthesize exclusively R-LPS. Such factors as bacterial growth phase and temperature influence LPS heterogeneity (6).

Each LPS region may induce synthesis of specific antibodies (Ab), able to activate the classical pathway (CP) of complement activation. However, in the absence of Ab, lipid A may activate CP *via* direct binding of C1, while core OS-LP (MBL-dependent) and O-PS may activate the alternative pathway (AP) and/or LP (involving MBL or ficolins) (7–10). Recently, MASP-1 (crucial for activating MASP-2 and therefore initiation of the LP cascade) was shown to participate in LPS-induced AP activation (11). Regarding core OS, l-*glycero*-d-*manno*-heptose (Hep) in the inner core region (characteristic for majority of LPS) and d-GlcNAc in the outer core region end (in *Salmonella enterica* serovar Minnesota) were reported as hMBL-binding motifs in R-LPS (12, 13). Although lipid A is considered the toxic principle of LPS, responsible for CD14–TLR-4–MD-2 complex-dependent immune cell response, the contribution of LPS polysaccharide-induced complement activation seems to be important for development of septic shock. Unlike lipid A-dependent endotoxic shock, polysaccharide-induced anaphylactoid reactions can be evoked in LPS-hyporesponsive mice (14, 15). Intravenous injection of certain S-LPS (but not isolated lipid A or R-type LPS) leads to rapid accumulation of platelets in the lungs and liver, followed by their degradation and release of serotonin, and death within 15–60 min, preceded by characteristic symptoms like convulsions and unconsciousness (16). Complement activated by LPS–MBL may be responsible for the degradation of platelets (16). LPS having mannose homopolymers (MHP) as O-PS (e.g., *Klebsiella pneumoniae* O3) (17) are potent inducers of anaphylaxis-like endotoxic shock in mice (16, 18). Some smooth bacteria (including *Proteus vulgaris* O25, *S. enterica* ser. Minnesota, and Abortusequi) have MBL-binding motifs within the core OS only and are capable of inducing a lethal early-phase shock (19, 20).

*Hafnia alvei* is an opportunistic human pathogen responsible for nosocomial mixed infections and sepsis (21). Most *H. alvei* LPS possesses smooth forms. So far, 40 O-serotypes (O-PS structures), and 4 types of core OS have been identified. *H. alvei* LPS is also an example of endotoxin having the *E. coli*-type structure of lipid A (22–24). A few strains of *H. alvei* synthesize LPS containing *E. coli* R4 [strains Polish Collection of Microorganisms (PCM) 23 or 1222] or *Salmonella* Ra (strain PCM 1212) core types (Figure 1) (25, 26). The OS1 hexasaccharide is the predominant core OS for this species, with Hep and Kdo residues in its inner core region like most Gram-negative bacteria (Table 1, footnote f) (24, 27).

**Table 1:** Structural characteristics of LPS and lectin blotting results of SDS-PAGE separated *Hafnia alvei* LPS with serum-derived hMBL.^a^

LPS	Region of interaction		Outer core OS motifs
	O-PS	Core OS/lipid A (core OS type)	
*Klebsiella pneumoniae* O3	+	+ (OS *K. pneumoniae*)	α-GlcN, α-Kdo, α-Hep^b^
*H. alvei* 23	−	− (R4)	nd
*H. alvei* 1190	+	+ (OS1^f^)	Hep-Kdo^c^(DS)
*H. alvei* 1192	−	+ (OS1^f^)	Hep-[Gal-]-Kdo^d^(TS1)
*H. alvei* 1196	+	+ (OS1^f^)	Gal-Hep-Kdo^e^(TS2)
*H. alvei* 1200	−	+ (OS1^f^)	Hep-Kdo^c^(DS)
*H. alvei* 1209	−	+ (OS1^f^)	Hep-Kdo^c^(DS)
*H. alvei* 1212	−	+ (Ra)	nd
*H. alvei* 1222	−	− (R4)	nd

*^a^All LPS represent smooth type molecules. “+” or “−” indicate positive or negative interaction with hMBL, respectively. R4, Ra, and OS1 indicate core oligosaccharides present in LPS of E. coli R4, Salmonella spp. Ra, and typical H. alvei core OS, respectively (26, 27)*.

*^b^Terminal residues present in outer core OS region (28)*.

*^c^l-α-d-Hepp3OAc-(1→4)-α-Kdop (10, 23, 29)*.

*^d^l-α-d-Hepp-(1→4)-[α-d-Galp6OAc-(1→7)]-α-Kdop (24)*.

*^e^α-d-Galp-(1→2)-l-α-d-Hepp-(1→4)-α-Kdop (30)*.

*^f^α-d-Glcp-(1→3)-α-d-Glcp-(1→3)-[l-α-d-Hepp-(1→7)]-l-α-d-Hepp4P-(1→3)-l-α-d-Hepp4PPEtn-(1→5)-α-Kdo (27)*.

*nd, not determined; LPS, lipopolysaccharide; hMBL, human MBL*.

*Schematic structures are presented in Figure 1*.

A peculiarity of *H. alvei* LPS is the presence of Hep-Kdo-containing motifs also in the outer core region (24) (Figure 1). Branched trisaccharide (TS1), l-α-d-Hep*p*-(1→4)-[α-d-Gal*p*6OAc-(1→7)]-α-Kdo*p*-(2→, was identified, for example, at the outer core region of *H. alvei* 32 and PCM 1192 LPS (OS1-TS1 core) (24, 31). Linear trisaccharide α-d-Gal*p*-(1→2)-l-α-d-Hep*p*-(1→4)-α-Kdo*p*-(2→ (TS2) is characteristic for *H. alvei* PCM 1196 (OS1-TS2 core) (32). The disaccharide (DS), l-α-d-Hep*p*3OAc-(1→4)-α-Kdo*p*-(2→ was identified in *H. alvei* PCM 1200 and 1209 (OS1-DS type core) (29). The presence of Hep-Kdo-containing motifs in the outer core region makes *H. alvei* LPS similar to *K. pneumoniae* and *P. vulgaris* O25 LPS (28, 33). This similarity prompted us to examine the ability of *H. alvei* LPS to bind MBL, activate human and murine complement systems and induce anaphylactoid reactions in mice.

Here, we explicate the structural basis of interactions between MBL and core OS of a variety of *H. alvei* LPS. These interactions lead to the activation of complement *via* the LP. Moreover, complexes of *H. alvei* LPS with MBL were able to induce anaphylactoid shock in BALB/c mice. LPS from 10 different species of opportunistic pathogens were tested to identify other examples of such interactions. We suggest that common interactions between core OS of LPS and MBL triggering LP activation might influence the course of Gram-negative infections, including nosocomial infections and sepsis. Therefore, consideration of surface antigen structure should be helpful in understanding pathogenicity and may influence development of new therapeutic strategies in Gram-negative sepsis.

## Materials and Methods

### Animals

BALB/c mice (males, 7–8 weeks old) were purchased from the animal facility of the Polish Mother’s Memorial Hospital, Research Institute, Lodz, Poland. The BALB/c mice were housed at the animal facility of the Ludwik Hirszfeld Institute of Immunology and Experimental Therapy (Wroclaw, Poland) and *in vivo* experiments were approved by the Local Ethical Commission for Animal Experimentation (Wroclaw, Poland).

### Bacteria

*Hafnia alvei* strains PCM 537, 1188, 1190, 1191, 1192, 1195, 1196, 1200, 1203, 1204, 1205, 1206, 1207, 1208, 1209, 1210, 1211, 1212, 1213, 1214, 1218, 1220, 1221, 1222, 1224, and *E. coli* O55 were obtained from the PCM at the Ludwik Hirszfeld Institute of Immunology and Experimental Therapy (Wroclaw, Poland). *Proteus* spp. strains (*P. mirabilis, P. vulgaris, P. penneri, P. myxofaciens*, and *P. genomospecies*) came from the collection of the Laboratory of General Microbiology, University of Lodz (Poland). *K. pneumoniae* O3:K55^−^ (strain 5505Δ*cps*) was kindly provided by Prof. S. Kaluzewski (National Institute of Hygiene, Warsaw, Poland). *H. alvei, E. coli*, and *K. pneumoniae* were grown till exponential phase (8 h) in Davis medium as described (34), and *Proteus* spp. strains were grown in liquid nutrient broth containing 1% glucose (35). They were stored in a glycerol mixture at −75°C.

### Sera

Sera obtained from BALB/c mice were used as a source of murine MBL and ficolins. Pooled normal human serum (NHS) was used as a source of hMBL and came from the collection of the Laboratory of Immunobiology of Infections, Institute of Medical Biology, Polish Academy of Sciences. Polyclonal rabbit sera anti-*H. alvei* core OS (OS1) conjugated with tetanus toxoid (OS1-TT) came from Laboratory of Microbial Immunochemistry and Vaccines (Ludwik Hirszfeld Institute of Immunology and Experimental Therapy, Wroclaw, Poland). Polyclonal rabbit immunoglobulins specific for TS (Hep-[Gal]-Kdo) were isolated from antisera using an adsorption on bacterial mass as previously described (24). DS-specific Ab were isolated by two-step affinity chromatography of anti-*H. alvei* 1209 serum (immunization with killed bacteria, DS-positive strain) on: (i) *H. alvei* 1209 core OS1-Sepharose 4B gel and (ii) *H. alvei* 1209 O-PS-Sepharose 4B gel. Both resins were prepared as previously described (10, 36, 37). Eluates containing anti-DS Ab were collected in sterile vials and stored at −20°C.

### Preparation of LPS

Lipopolysaccharide were extracted from bacterial cells by the hot phenol/water method (38) and purified by ultracentrifugation as previously described (34, 35). *Proteus* spp. LPS were extracted from dried bacterial cells, as previously described (39), by the phenol–water procedure according to the method of Westphal and Jann (38) and purified with aqueous 50% trichloroacetic acid. For analyses of growth phase dependence of hMBL–bacteria interactions (SDS-PAGE and lectin blotting), LPS was isolated from bacteria by Tri-Reagent method (40). LPS of *H. alvei* strains 1, 2, 17, 23, 31, 32, 37, 38, 39, 114/60, 481L, 600, 744, 981, *Edwardsiella anguillimortifera, Citrobacter* (kindly provided by Prof. E. Katzenellenbogen), and *E. coli* came from the collection of the Laboratory of Microbial Immunochemistry and Vaccines (Ludwik Hirszfeld Institute of Immunology and Experimental Therapy, Wroclaw, Poland).

### O-PSs, Core Oligosaccharides, and Lipid A Isolation

Polysaccharides, oligosaccharides, and lipids A were isolated by mild acidic hydrolysis of *H. alvei* PCM 1190, 1192, and 1200 LPS at 100°C for 45 min. Poly- and oligosaccharides were fractionated and purified as previously described using Bio-Gel P-10 (10). The Hep-Kdo-containing fraction was isolated from the heterogeneous core OS fraction and analyzed by the use of liquid chromatography-electrospray ionization-tandem mass spectrometry (LC-ESI-MS) on SeQuant^®^ZIC^®^-HILIC column as previously described (41). Fractions 3 and 4 were pooled and used for surface plasmon resonance (SPR) analysis. Lipid A was isolated as a water-insoluble fraction of the LPS hydrolyzate. Prior to lectin blotting, lipids A were purified by extraction with 2:1:3 chloroform/methanol/water mixture (v/v/v) to remove membrane phospholipids and remains of LPS. Both water phase (w) and chloroform (ch) phase lipids A were collected (23).

### SDS-PAGE

The LPS and lipids A were analyzed by SDS-PAGE. Briefly, 4.5 and 15.4% polyacrylamide-bisacrylamide gels were used as the stacking and resolving gels, respectively. Glycolipids (3 µg) were mixed with sample buffer (65 mM Tris, pH 6.8, 2% SDS, 35% glycerol, 0.6 M DTT, ~0.1% bromophenol blue) in ratio 1:1 (v/v). LPS/lipid A bands were visualized by the silver staining method (42).

### Lectin Blotting

SDS-PAGE-separated LPS were transferred onto polyvinylidene fluoride membranes (Bio-Rad, USA). Membranes were blocked with SuperBlock^®^ Blocking Buffer (Thermo Scientific, USA) for 2 h, followed by overnight incubation at 4°C, with 25-fold diluted human or murine serum as previously described (10). Bound proteins were detected by immunostaining with different primary Ab: (i) monoclonal mouse anti-hMBL Ab (clone HYB 131-01, BioPorto, Denmark), (ii) monoclonal rat anti-MBL-A (clone 2B4) and (iii) anti-MBL-C Ab (clone 16A8) (both from Hycult Biotech, The Netherlands), (iv) rabbit anti-ficolin-A kindly provided by Dr. Yuichi Endo (Fukushima Medical University, Fukushima, Japan), and (v) reactions were detected with HRP-conjugated rabbit anti-mouse, anti-rat secondary IgG Ab (Dako, Denmark) or anti-rabbit IgG secondary Ab, and visualized with Immun-Star HRP Chemiluminescent Substrate Kit (Bio-Rad, USA) and G:Box chemiluminescent imaging system (Syngene, UK). Nonspecific interactions of secondary Ab were excluded by controls without the primary Ab or the serum as a source of hMBL.

### ELISA for Lectin Binding

Interactions of hMBL, MBL-A, MBL-C, and murine ficolins A and B with *H. alvei* LPS were tested as previously described (10). Briefly, NUNC Maxisorp U96 plates were coated with 2 µg of LPS/well. After blocking with 0.1% BSA in TBS-Ca^2+^ buffer (10 mM Tris, 120 mM NaCl, 1 mM CaCl_2_, pH 7.4), NHS or murine serum (prediluted in 0.1% BSA/20 mM Tris, 1 M NaCl, 10 mM CaCl_2_, pH 7.4) was added. After overnight incubation at 4°C, the bound proteins were detected by using specific primary antibodies (mentioned in the lectin blotting procedure) and HPR-conjugated anti-mouse, anti-rat, or anti-rabbit corresponding anti-IgG antibodies (Dako, Denmark). As substrate for peroxidase, 2,2′-azino-bis(3-ethylbenz-thiazoline-6-sulfonic)acid (ABTS) (Sigma, USA) was employed. Absorbance values were measured at 405 nm using Benchmark Plus microplate spectrophotometer (Bio-Rad).

### Determination of hMBL–MASP-2 and MBL–MASP-1 Complex Activity

Activity of lectin(s)-MASP-2 complexes was determined as previously described (43) with modification (44). LPS from various bacteria were used for coating of microtiter plates (Maxisorp U96, Nunc). The products of C4 activation were detected with rabbit anti-hC4c and HRP-conjugated anti-rabbit Ig (Dako). To test MBL-MASP-1 complex activity, VPR-AMC (Val-Pro-Arg-aminomethylcoumarin) peptide (Bachem, Switzerland), as the substrate for MASP-1 was used as previously described (45) and the fluorescence was read using a Varioskan Flash reader (Thermo Scientific, USA).

### Flow Cytometry Analysis of Binding of hMBL to Formaldehyde-Inactivated Bacteria

Flow cytometry analysis was performed as previously described (10). Depending on experiment, bacteria were cultured and harvested at lag phase (3 h), log phase (6 h), or stationary phase (24 h) of growth. The growth phase of culture was determined on the basis of optical density measurement at 600 nm and the appropriate growth curve. Immediately before each experiment, bacterial cells were centrifuged, washed with PBS, and suspended in 10-fold diluted NHS (pool), used as a source of hMBL. Monoclonal anti-hMBL Ab (clone HYB 131-01) and fluorescein isothiocyanate-labeled anti-mouse IgG Ab (Dako) were used as detection system. The analysis of the FITC-labeled bacteria was performed using a Cytomics FC 500 MPL Beckman-Coulter (USA) flow cytometer. Bacteria were detected using log-forward and log-side scatter dot plot. Gating region was set to exclude debris and larger aggregates of bacteria. A total of 10,000 events were acquired.

### Induction of Anaphylaxis-Like Endotoxic Shock in muramyldipeptide (MDP)-Primed Mice

The BALB/c mice were treated i.p. with 100 µg of MDP in PBS (20), and after 4 h, animals received i.v. 100 µg of LPS. *K. pneumoniae* O3 and *E. coli* O55 LPS were used as a positive and negative control, respectively. Incidence, severity, and scoring of the anaphylaxis-like shock were recorded within 30 min: 0, no signs of shock; 1, staggering; 2, crawling and prostration; 3, prostration and weak convulsions; 4, prostration and strong convulsions (16). Subsequent mortality was recorded within 1 h and after 24 h after LPS injection.

### Surface Plasmon Resonance

Surface plasmon resonance studies were assessed with a Biacore T200 system (GE Healthcare Bio-Science AB, Sweden). Carrier free recombinant hMBL (R&D Systems, USA) was immobilized in 10 mM sodium acetate, pH 4.0 on the CM5 series S sensor chip (GE Healthcare Bio-Science AB) at a flow rate 5 µl/min, to the level of 16,000 RU using the amine coupling chemistry. A flow cell with immobilized 240 RU of ethanolamine was used as a reference surface. HBS-P buffer (GE Healthcare Bio-Science AB) supplemented with Ca^2+^, Mg^2+^ ions (5 mM MgCl_2_, 5 mM CaCl_2_) was used as a running buffer. *H. alvei* O-PS (PCM 1190, 1196, 1200, and 1209) and core hexasaccharides from *H. alvei* PCM 1200 LPS at various concentrations were injected at a flow rate 30 µl/min. 0.5% SDS injected for 30 s was used as a regenerator in all SPR experiments.

## Results

### *H. alvei* LPS Core Oligosaccharide Is a Common MBL Target

**Figure 2:**
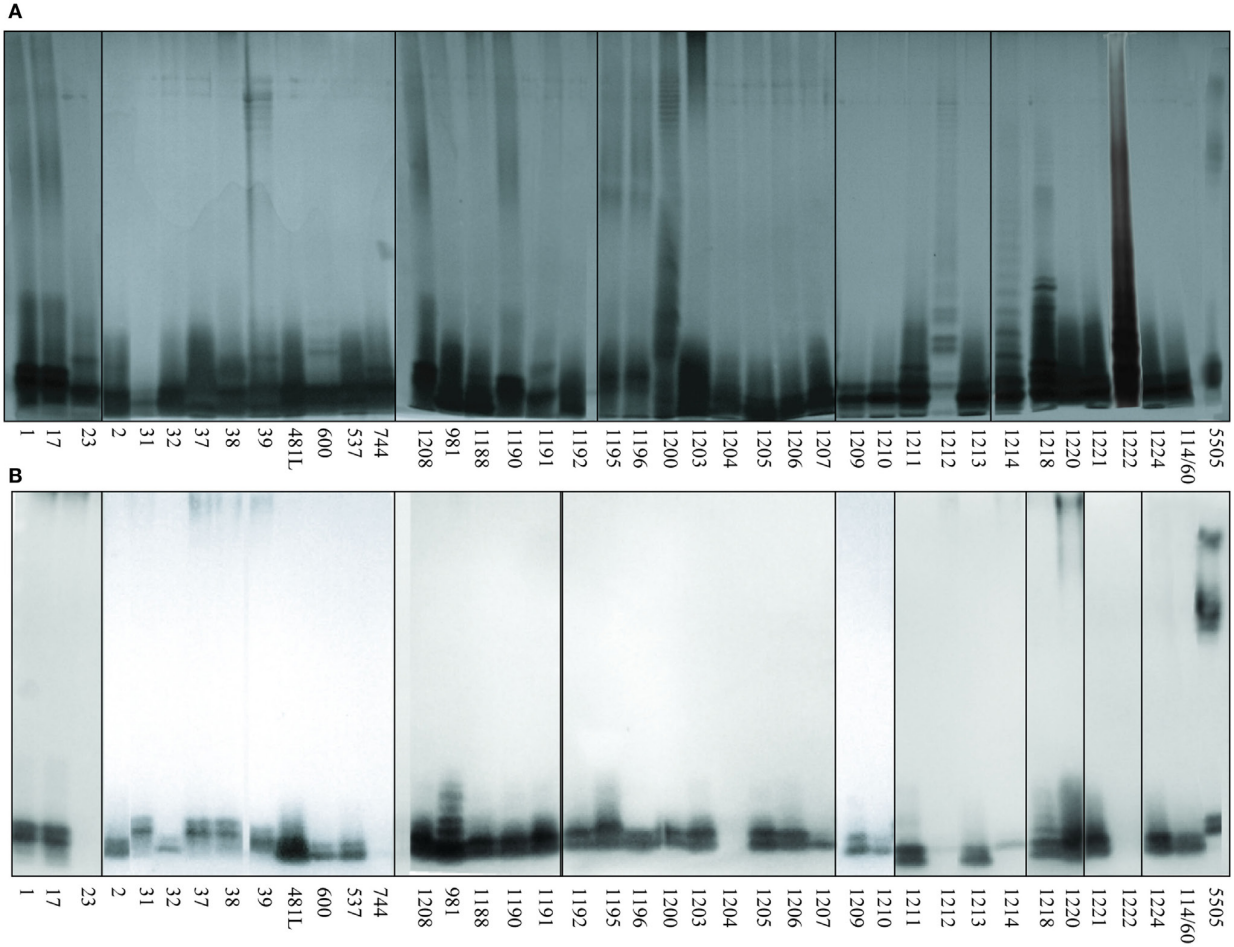
Silver stained SDS-PAGE **(A)** and lectin blotting analysis **(B)** of the interaction of human MBL (hMBL) with *Hafnia alvei* lipopolysaccharides (LPSs). Normal human serum (pool) was used as a source of hMBL. Lanes are depicted with the strain number in Polish Collection of Microorganisms. The number 5505 represents *Klebsiella pneumoniae* O3:K55^−^ LPS used as a positive control.

Screening for the presence of structural motifs recognized by hMBL was performed by lectin blotting for LPS isolated from 39 different O-serotypes of *H. alvei* (Figure 2). LPS isolated from smooth strains gave a characteristic ladder-like multi-band pattern after SDS-PAGE reflecting natural heterogeneity of LPS on the cell wall surface and facilitating core OS accessibility (Figure 2A). The fast-migrating fractions originate from lipid A substituted with core OS, whereas the slow-migrating fractions show the length distribution of the polymer built up of lipid A-core OS substituted with varying numbers of O-PS repeating units. LPS isolated from *K. pneumoniae* O3 strain 5505 (MHP as O-PS) was used as a positive control, where interactions were observed both in the core OS/lipid A and O-PS regions. hMBL recognized 34 out of 39 *H. alvei* LPS. Generally, reactivity was related to the fast-migrating fractions of the core OS-lipid A region. No interactions were observed for *H. alvei* 23, PCM 744, 1204, 1212, and 1222 LPS (Figure 2B).

**Figure 3:**
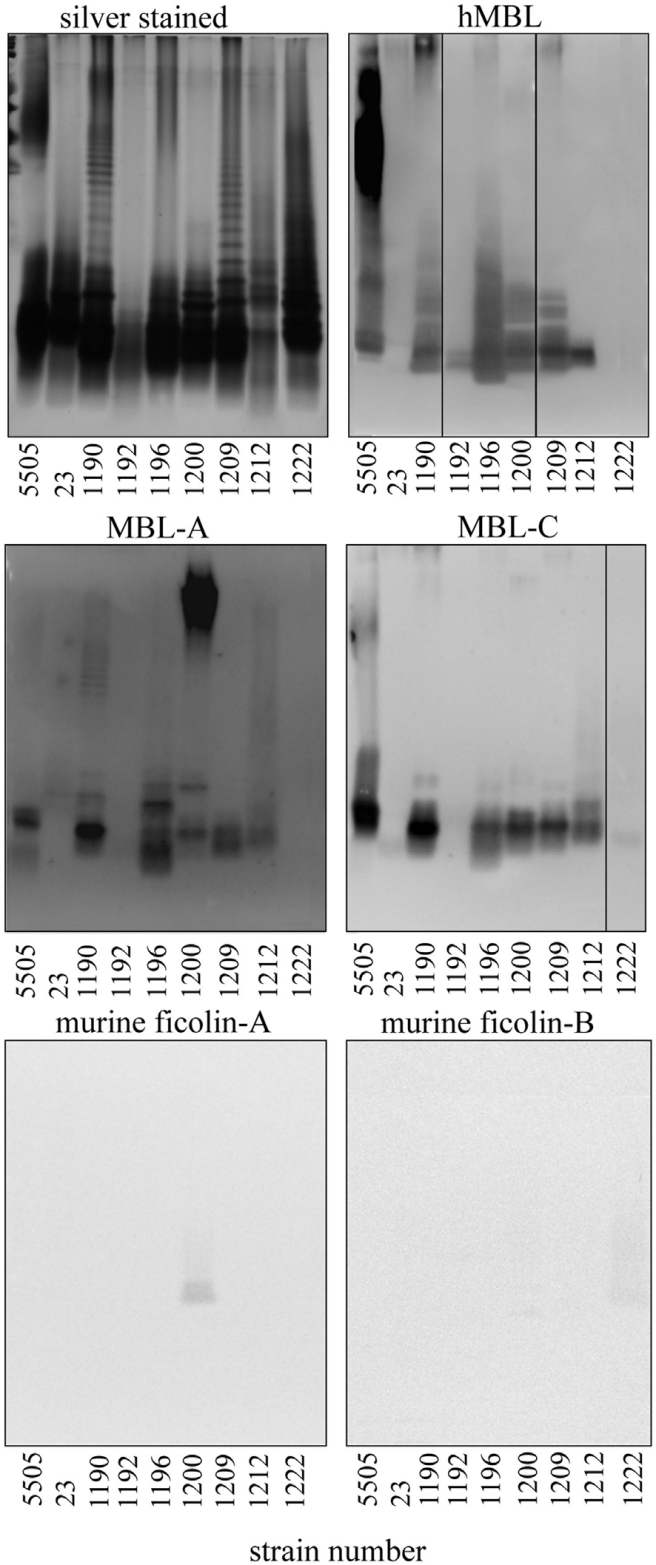
Silver stained SDS-PAGE of representative *Hafnia alvei* lipopolysaccharides (LPSs) and lectin blotting of their interactions with human MBL (hMBL) and murine mannose-binding lectin (MBL)-A, MBL-C, ficolin-A, and ficolin-B. Normal human serum and BALB/c mice sera were used as a source of hMBL or murine MBL-A, MBL-C, ficolin A, and B, respectively. Lanes are depicted with the strain number in Polish Collection of Microorganisms. Strain 5505 represents *Klebsiella pneumoniae* O3:K55^−^ LPS used as a positive control.

**Figure 4:**
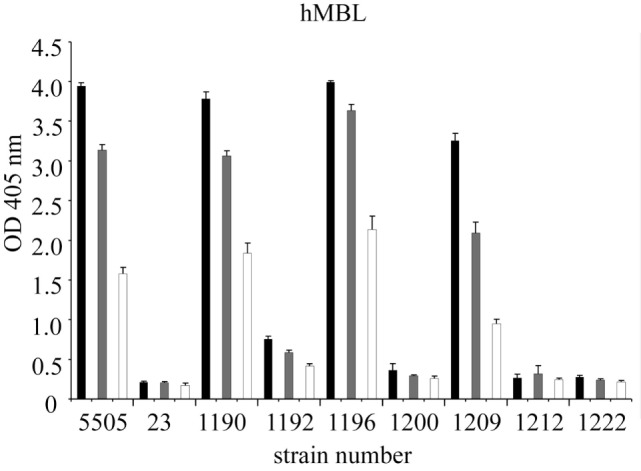
Recognition of selected *Hafnia alvei* lipopolysaccharides (LPSs) by human MBL. Microtiter plates were coated with LPS, incubated with NHS to measure interactions with hMBL. Black, gray, and white bars represent 200-, 400-, and 800-fold dilutions, respectively. Data represent the mean values of four replicates and are marked by PCM strain number. Strain 5505 represents *Klebsiella pneumoniae* O3:K55^−^ LPS used as a positive control. Abbreviations: OD, optical density measured at λ = 405 nm; hMBL, human MBL; NHS, normal human serum; PCM, Polish Collection of Microorganisms.

Nine representative LPS, chosen on the basis of well-characterized structure (Figure 1) and different hMBL-binding patterns (Figure 2B; Table 1), were selected for further experiments to explore human and murine MBL specificity (Figure 3). For *H. alvei* PCM 1192, 1200, 1209, and 1212, hMBL bound within the core OS region only. For *H. alvei* PCM 1190 and 1196, hMBL bound within both the core OS and O-PS regions. For *H. alvei* 23 and PCM 1222, no binding was observed. These LPS–hMBL interactions were confirmed by ELISA (Figure 4). Eight *H. alvei* LPS (and *K. pneumoniae* O3 LPS as positive control) were used as solid-phase antigens. The strongest reactions of serum hMBL were observed for LPS *K. pneumoniae* O3 and *H. alvei* PCM 1190, 1196, and 1209 LPS. In contrast to the lectin blotting, no reaction with *H. alvei* PCM 1200 LPS was observed, what might be explained by competition between strong binding of O-PS-reactive ficolin-3 (10) and moderate binding of core OS-reactive hMBL. In addition, long O-PS chains of LPS 1200 might also hinder hMBL access to core OS.

**Figure 5:**
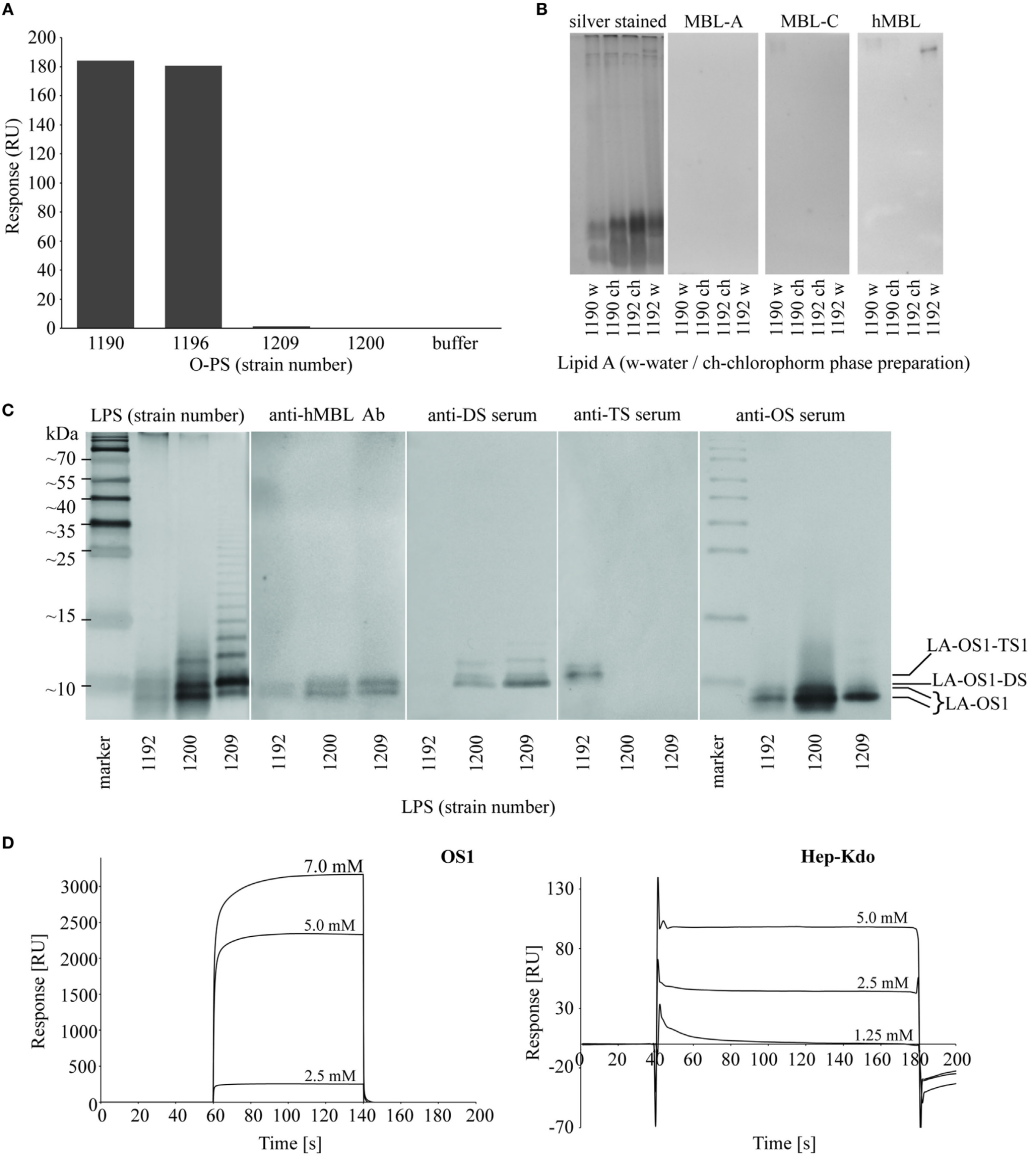
Identification of hMBL-binding regions in *Hafnia alvei* lipopolysaccharides (LPSs). **(A)** Surface plasmon resonance (SPR) analysis of interactions between recombinant hMBL and O-specific polysaccharides (O-PSs) of *H. alvei* PCM 1190, 1196, 1200, and 1209 LPS. The bars represent binding response of immobilized recombinant hMBL (immobilization level: 16,000 RU) to O-PS (0.5 mg/ml) in running buffer HBS-P supplemented with MgCl_2_ and CaCl_2_. **(B)** Silver stained SDS-PAGE and lectin blotting analysis of the interaction between *H. alvei* lipids A [water phase (w) and chloroform phase (ch) preparations] and serum-derived murine MBL-A, MBL-C, and hMBL. Lanes are depicted with the strain PCM number. **(C)** Silver stained SDS-PAGE and lectin blotting analyses of the interactions between LPS of *H. alvei* PCM 1192 (TS1-positive), 1200 (DS-positive), 1209 (DS-positive) and serum-derived hMBL, DS-specific rabbit serum, TS1-specific rabbit serum, and OS1-specific rabbit serum. M—molecular weight markers. Marked bands corresponded to different forms of R-LPS: LA-OS1, LA-OS1-DS, LA-OS1-TS1, where LA stands for lipid A. **(D)** SPR sensograms for interactions of recombinant hMBL and OS1 and Hep-Kdo-containing OS isolated from *H. alvei* 1200 LPS. Sensor chip: CM5; Abbreviations: RU, response units; hMBL, human MBL; PCM, Polish Collection of Microorganisms; MBL, mannose-binding lectin.

Since murine model was chosen for further studies to test *in vivo* activity of LPS on complement-mediated anaphylaxis-like endotoxic shock, the reactivity of LPS with murine MBL-A and MBL-C (as well as with ficolin A and ficolin B) was analyzed by lectin blotting (Figure 3). Murine MBL-C showed binding pattern very similar to hMBL within the core OS region. Strong reactions with *H. alvei* PCM 1190, 1196, 1200, 1209, 1212, and *K. pneumoniae* O3 LPS and negligible reactions with LPS 23, PCM 1192 and 1222 were noted. In addition, interactions with high molecular weight fractions (O-PS region) were easily visible for *H. alvei* 1190 and *K. pneumoniae* O3 LPS. For MBL-A, no reactivity was observed for PCM 1192 and 1222 and very weak reactivity for 23 (within O-PS). Binding of this lectin to LPS *K. pneumoniae* O3 and PCM 1209 strains was attributed to core OS only. Reactivity within both core OS and O-PS region were observed for *H. alvei* PCM 1190, 1196, 1212, and 1200. Especially strong recognition of PCM 1200 LPS was unique for MBL-A, whereas MBL-C (similarly to hMBL) were devoid of such activity. Moreover, only traces of signal related to O-PS region were observed for interactions of ficolin A with PCM 1200 and ficolin B with PCM 1222 (Figure 3). Interactions of MBL-A and MBL-C with *H. alvei* lipid A was excluded by lectin blotting (Figure 5B).

### Hep-Kdo Motifs in *H*. *alvei* LPS Inner and Outer Core Are Recognized by MBL

From lectin blotting (Figures 2 and 3), it was suggested that most *H. alvei* LPS were bound by hMBL *via* the core OS/lipid A region. SPR analyses confirmed interactions of hMBL with O-PS regions of PCM 1190 (46) and 1196 (47) and excluded O-PS of PCM 1209 and 1200 LPS as targets for the lectin (Figure 5A). Data from lectin blotting with the use of purified lipid A fractions of *H. alvei* PCM 1190 and 1192 confirmed the lack of hMBL reactivity with that part of LPS (Figure 5B).

Next, we identified the core OS regions involved. Immunostaining with the use of OS1, DS- and TS1-specific Ab revealed four bands of low molecular weight fractions of migrating LPS (Figure 5C) attributed to lipid A-OS1 (two bands), lipid A-OS1-TS1, and lipid A-OS1-DS molecules. Two bands marked by lipid A-OS1 reflected OS1 heterogeneity related to ethanolamine, phosphate groups, and glycine substituents and are common for all three studied LPS (PCM 1192, 1200, and 1209). The band assigned as lipid A-OS1-DS was present in DS-expressing LPS of *H. alvei* PCM 1200 and 1209, while the lipid A-OS1-TS1 band in LPS of *H. alvei* PCM 1192 LPS.

The ability of recombinant hMBL to bind different core OS fractions of *H. alvei* LPS was further investigated by SPR on Biacore T200 (Figure 5D). Core OS isolated from PCM 1200 LPS were used as analytes. Both isolated OS1 and low molecular weight fraction of Hep-Kdo interacted with immobilized recombinant hMBL in a concentration-dependent manner, with higher affinity observed for OS1. It was also confirmed by ELISA inhibition assay with the use of both analytes (data not shown).

### Bacterial Growth Phase Determines the Accessibility of LPS Core Region for MBL

**Figure 6:**
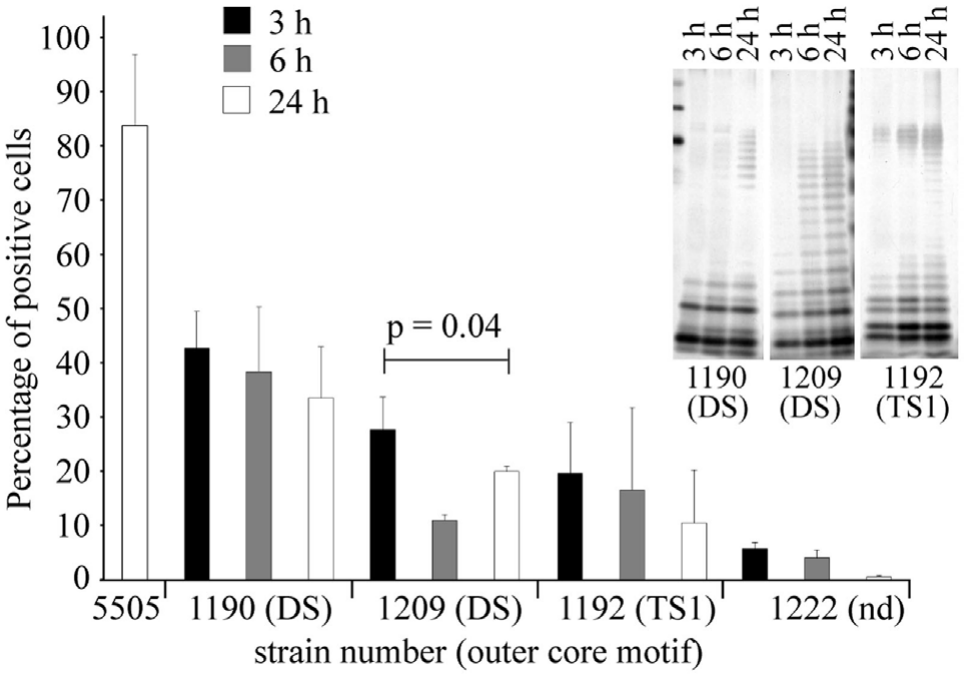
Growth phase-dependence of hMBL–*Hafnia alvei* interactions observed in FACS analyses. Binding of hMBL to inactivated smooth *H. alvei* PCM 1190, 1192, and 1209 bacterial cells at the lag (black bars), exponential (gray bars), and the stationary phases (white bars) of growth. Silver stained SDS-PAGE (inset) of LPS 1190, 1209, and 1192 isolated from bacterial mass collected at lag, log, and stationary phases. Data represent the mean values ± SD from three independent experiments. DS and TS1 stand for the disaccharide and trisaccharide outer core motif in LPS of selected *H. alvei* strains. Abbreviations: Nd, not determined; hMBL, human MBL; PCM, Polish Collection of Microorganisms; LPS, lipopolysaccharide.

Binding of hMBL to LPS on bacterial surface was further investigated by flow cytometry. Since bacterial growth phase may be associated with changes in LPS expression, accessibility of core OS regions for hMBL was examined using microbial cells collected at lag (3 h), log (6 h), and stationary phase (24 h) (Figure 6). Four strains were chosen for this study: (i) *H. alvei* PCM 1190 recognized by hMBL within DS-carrying core OS and O-PS regions, (ii) *H. alvei* PCM 1192 and 1209 with hMBL targets located in low molecular weight fraction of LPS (core OS1 decorated with TS1 or DS, respectively), and (iii) *H. alvei* PCM 1222 expressing LPS not recognized by hMBL. *K. pneumoniae* O3 grown to the stationary phase was used as a positive control. The percentage of hMBL-labeled cells was confronted with R-LPS and S-LPS distribution examined by SDS-PAGE analysis of LPS extracted from cells at lag, log, and stationary phases (Figure 6, inset).

For all hMBL-reacting strains (PCM 1190, 1192, and 1209), the highest values of labeled cells were recorded for lag phase, where R-LPS represented the prevailing LPS population. The low content of O-PS chains facilitated access of hMBL to outer core regions of R-LPS (OS1 and DS). For strains PCM 1190 and 1192, the proportions of labeled cells were inversely associated with the expression of the S-LPS population. The highest values, at each growth phase, were recorded for the PCM 1190 strain. This was expected since its LPS has MBL-binding motifs not only in the core but also in O-PS region. PCM 1192 and 1209 LPS were recognized by hMBL within the core OS region only, whereby the most efficient binding was observed for bacteria at the lag phase (10.4 and 27.7% positive cells, respectively). In contrast to other strains, PCM 1209 bacteria showed the lowest accessibility for hMBL at log but not stationary phase. That might be explained by a higher content of R-LPS forms with accessible OS1 and DS motifs at stationary phase contrary to log phase (as evidenced by SDS-PAGE). Performed experiments demonstrated that observed relationships clearly resulted from LPS structure, i.e., the length of O-PS chains that hindered structural motifs recognized by hMBL (OS1 and DS).

### Interaction of hMBL With *H. alvei* LPS Leads to Complement Activation

**Figure 7:**
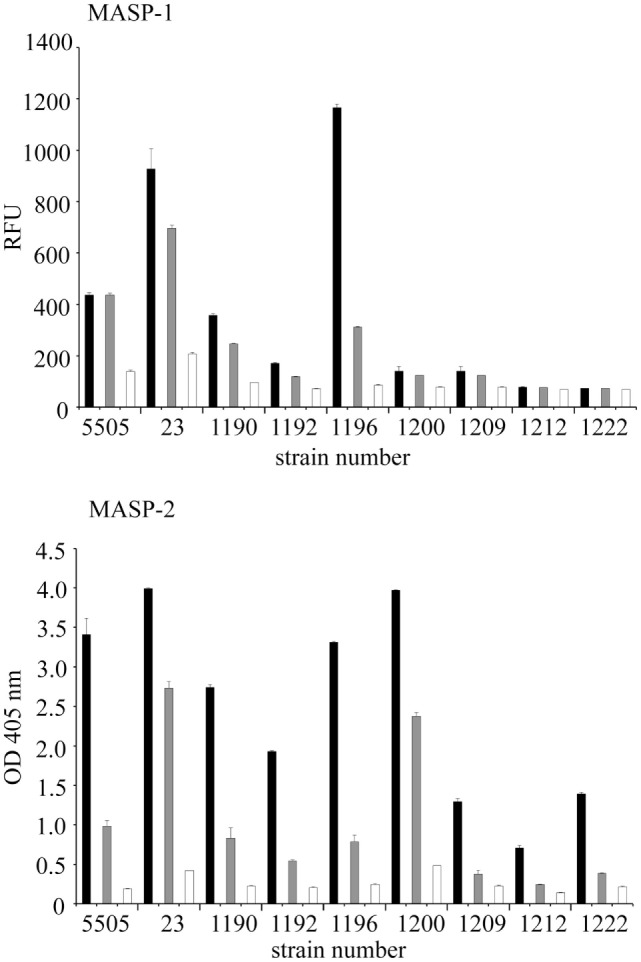
Activation of the human lectin pathway of complement by *Hafnia alvei* LPS. (Top) Human LPS-MASP-1 activity (ability to cleave a VPR-AMC peptide). Black, gray, and white bars represent 5-, 20-, and 40-fold dilutions, respectively. (Bottom) Human LPS–MASP-1/2 complex-dependent C4c deposition. Black, gray, and white bars represent 50-, 200-, and 800-fold dilution, respectively. Each dilution series is described by the strain number on the *X*-axis. Pattern recognition molecule states for MBL or other complement-activating collectin/ficolin present in serum. Data represent the mean values of four replicates. LPS were used as solid phase. Abbreviations: RFU, relative fluorescence units; OD, optical density measured at λ = 405 nm; LPS, lipopolysaccharide; MBL, mannose-binding lectin; MASP, MBL-associated serine proteases.

The ability of selected LPS to initiate the complement cascade *via* the LP was tested by investigating activation of MASP-1 (cleavage of synthetic substrate, VPR-AMC) and MASP-2 (cleavage of C4) dependent on LPS recognition by LP molecules, especially hMBL. MBL–MASP-1 concentration-dependent activation was triggered by *H. alvei* 23, PCM 1190, 1192, 1196, 1200, 1209 LPS, as well as *K. pneumoniae* O3 (control) (Figure 7). The deposition of C4 activation products was additionally noted for PCM 1212 and 1222 LPS. It is worth mentioning that procedure employed does not exclude activation of LP by complexes of ficolin-3 with MASP (as described previously for 23 and PCM 1200 LPS) (10) or other than MBL collectins. Contribution of ficolin-1 and -2 was excluded (10). The influence of CP was excluded by high ionic strength of the buffer that inhibits the binding of C1q to immune complexes and disrupts the C1 complex, whereas MBL complexes integrity is maintained (48). The variations in reactivity profiles (Figures 4 and 7) may reflect differences in serum dilution used and sensitivity of assays.

### Interaction of MBL With *H. alvei* LPS Core Oligosaccharide Induces Anaphylactoid Shock

The biological consequences of *in vivo* MBL interaction with *H. alvei* PCM 1190, 1192, 1200, 1209, and 1212 LPS were tested by ability to induce an anaphylactoid reaction in mice. *K. pneumoniae* O3 and *E. coli* O55 as well as *H. alvei* PCM 1222 LPS were used as positive and negative controls, respectively (14).

Intravenous injection of *H. alvei* PCM 1190, 1200, 1209, or 1212 LPS-induced rapid shock (within 30 min, score 3–4) leading to death of MDP-sensitized BALB/c mice (Table 2). The distinctive effect was observed for LPS of *H. alvei* PCM 1200 that was strongly recognized by MBL-A within the O-PS region and moderately within the OS1-DS core region (Figure 3). The reaction, comparable to that provoked by *K. pneumoniae* O3 LPS, was also induced by *H. alvei* PCM 1190 (OS1-DS core type) and 1212 (*Salmonella* Ra core type) moderately bound by MBL-A and MBL-C within the O-PS and core OS regions. *H. alvei* PCM 1209 LPS (OS1-DS core type) that was reactive for murine MBL-A and MBL-C within the core OS region only (Figure 3), still had a powerful ability to induce an anaphylactoid shock similar to *K. pneumoniae* O3. By contrast, mice treated with *H. alvei* PCM 1192 (OS1-TS1 core type) or 1222 (*E. coli* R4 core type) LPS developed mild or no characteristic symptoms within the first hour and died in the late phase of endotoxic shock (lipid A-dependent) (Table 2), similar to animals injected with *E. coli* O55 LPS (negative control).

**Table 2:** Ability of LPS from smooth strains of *Hafnia alvei, E. coli*, and *Klebsiella pneumoniae* to induce anaphylactoid shock and death in BALB/c mice.^a^

LPS (core OS type)	LPS region recognized by		Shock^b^		
	MBL-A	MBL-C	Incidence	Score^c^ 30 min	Mortality
*H. alvei* 1200 (OS1-DS)	O-PS/core OS	Core OS	5/5	10 min: 4	0.5 h: 5/5
*H. alvei* 1209 (OS1-DS)	Core OS	Core OS	5/5	3–4	0.5 h: 4/5
					1 h: 5/5
*H. alvei* 1212 (Ra)	O-PS/core OS	Core OS	4/4	3–4	0.5 h: 4/4
*H. alvei* 1190 (OS1-DS)	O-PS/core OS	Core OS	5/5	3	0.5 h: 5/5
*K. pneumoniae* O3^d^	Core OS	O-PS/core OS	5/5	3–4	0.5 h: 5/5
*H. alvei* 1192 (OS1-TS1)	–	–	4/4	0–1	0.5 h: 1/4
					24 h: 4/4
*H. alvei* 1222 (R4)	–	–	5/5	0–1	0.5 h: 0/5
					24 h: 5/5
*E. coli* O55	–^e^	–^e^	0/4	0	0.5 h: 0/4
					24 h: 4/4

*^a^Mice were sensitized with 100 µg of muramyldipetide (i.p.) and 4 h later injected with 100 µg of LPS (i.v.). Incidence, score, and mortality were recorded within 0.5 and 1 h after LPS injection*.

*^b^Incidence and mortality are shown as number/total*.

*^c^The scoring of anaphylaxis-like endotoxic shock was as follows: 1, staggering; 2, crawling and prostration; 3, prostration and weak convulsions; 4, prostration and strong convulsions (16)*.

*^d^The O-PS of this LPS is composed of mannose homopolymer*.

*^e^Determined only by inhibition of murine MBL–LPS O55 interaction in ELISA with LPS O55*.

*LPS, lipopolysaccharide; MBL, mannose-binding lectin*.

### LPS Core Oligosaccharide Is a Common MBL Target in Many Gram-Negative Bacteria

Screening for LPS from a variety of opportunistic pathogens (Table S1 in Supplementary Material), recognized by serum hMBL was performed with the use of lectin blotting. False positive reactions of primary and secondary detecting Ab were excluded. We found interactions between hMBL and LPS core regions to be very common: 13 of 15 *K. pneumoniae*, 11/22 *P. vulgaris*, 10/33 *P. mirabilis*, 7/15 *P. penneri*, 1/1 *P. myxofaciens*, 5/10 *E. coli* (including R-LPS containing R2 and R3 core types), 1/5 *Citrobacter* spp., and all of 4/4 *Edwardsiella anguillimortifera* LPS gave positive results.

## Discussion

Lipopolysaccharide is a major pathogen-associated molecular pattern (PAMP) and virulence factor of Gram-negative bacteria, responsible for development of sepsis and septic shock. Whereas the role of lipid A in those life-threatening events is well-documented (49), the influence of the polysaccharide region is poorly characterized. It is known that the carbohydrate moiety influences endotoxin clearance and biological activity of lipid A (50, 51). Recognition of LPS polysaccharide by a variety of pattern recognition molecules may lead to complement activation *via* CP, AP, and/or LP, all involved in sepsis development (52).

The core OS-lipid A region is a target for such plasma proteins as LPS-binding protein, BPI (bactericidal/permeability-increasing protein), CAP18 (cationic antimicrobial protein), and lysozyme. Consequently, bactericidal and inflammatory processes are induced by the host immune system. Due to the high structural heterogeneity of O-PS, the number of innate immunity factors interacting with that region is much lower. One example is ficolin-3, recognizing *H. alvei* PCM 1200 O-PS resulting in LP activation (10). Ficolin-3 was also demonstrated to enhance agglutination, phagocytosis, and killing of *H. alvei* PCM 1200 bacteria (53). Another example is pulmonary surfactant collectin SP-D binding mannose-rich O-PS of *K. pneumoniae* O3 and O5 (54).

This study provided well-documented evidence that core OS is the main target for human and murine MBL. Depending on the assay, the binding of recombinant (SPR) or NHS or murine serum MBL (lectin blotting, ELISA, flow cytometry) was detected in presented studies. Thus, it is worth noting that the oligomer distribution may vary for recombinant and NHS-derived MBL according to purification procedure (55), what may influences binding affinity between MBL and target ligand. Notwithstanding similar oligomer distribution patterns were reported for both forms (55, 56), including trimeric and tetrameric forms. Even though proposed oligomerization models indicated a polypeptide dimer as the basic unit in this process for MBL (57), higher oligomeric states are usually detected in rMBL and NHS-derived MBL preparations that ensure complement activation (56).

Performing screening analysis, we have shown that interactions of serum hMBL with different core OS regions were prevalent among LPS isolated from numerous opportunistic pathogens, such as *H. alvei, E. coli, K. pneumoniae, Proteus* spp., *Citrobacter* spp., and *E. anguillimortifera* representing different O-serotypes (O-PS structure) (Table S1 in Supplementary Material). Among 145 LPS tested, as much as approximately 57% were recognized by hMBL within fast-migrating fractions (corresponding to R-LPS built up of lipid A-core OS), whereas the reaction with slow-migrating fractions (S-LPS containing O-PS) was found in approximately 10% only. Accordingly, hMBL interacted with 34 (approximately 87%) out of 39 *H. alvei* LPS within the core OS region of LPS. Moreover, we also showed that highly purified *H. alvei* hexaacylated lipid A is recognized neither by human nor murine MBL. However, such interactions were previously suggested by Ono et al. (58) and Shiratsuchi et al. (59) for commercially available *E. coli* lipid A. This discrepancy may result from impurities of bacterial origin in the commercial preparations or the presence of MBL-binding motifs other than in the lipid A region of LPS (residuals of complete LPS after lipid A isolation by LPS hydrolysis).

Clinical isolates of Gram-negative bacteria are commonly of smooth type and therefore synthesize a highly heterogeneous pool of LPS, consisting of long-chain S-LPS, shorter S-LPS, and R-LPS unsubstituted by O-PS. We found that *H. alvei* PCM 1209 core OS within R-LPS forms exposed on the bacterial surface is accessible for hMBL (Figure 6). SPR analysis (Figure 5A) clearly demonstrated that PCM 1209 O-PS is not the MBL target. The core OS accessibility may depend on natural LPS heterogeneity (coexistence of R-LPS and S-LPS in smooth strains), and is hindered by core OS substitution with O-PS. It may be influenced by growth phase or environmental conditions. Generally, expression of R-LPS containing hMBL-binding motifs decreased with the culture progression (from lag to stationary phase) (Figure 6). Moreover, the immune response against O-PS may cause selective pressure on bacteria to lose the ability to express it (phase variation) (60, 61).

The LPS core OS region is relatively conservative and usually composed of an inner core and an outer core built up of Kdo and Hep residues and hexoses and hexosamines, respectively. For example, among *Salmonella* spp. strains one prevailing core type was described (Ra). Using mutants with defects in LPS core OS synthesis it was demonstrated that Hep residues in the inner core region are recognized by human and murine MBL due to their accessibility in truncated and incomplete core OS (12). Even though the inner core is common for the majority of enterobacterial LPS and represents MBL-binding motifs, our results indicated also outer core structures as natural MBL ligands. Hep and Hep-Kdo motifs were detected also in the latter region, for example, in *P. vulgaris* O25 and *K. pneumoniae* O3, O1, O2, O4, and O5 LPS (28, 33) as well as in numerous *H. alvei* strains (expressing DS, TS1, and TS2) (Figure 1). The lectin blotting (Figure 5C) and SPR analysis (Figure 5D) revealed recombinant MBL binding to DS-decorated *H. alvei* PCM 1200 OS1 and OS1 alone. Moreover, interaction of MBL with purified Hep-Kdo-containing motifs was also evidenced (Figure 5D), and determined by Hep residue (but not Kdo) according to the previous reports (12, 62). In spite of *manno* configuration, Kdo residues (even terminal) might be excluded as an MBL ligand, since deep rough mutants (Re) of *S. enterica* ser. Typhimurium or *Yersinia enterocolitica* O3, expressing LPS consisting of lipid A and one, two or three Kdo residues were not recognized (12, 63). Thus, Hep and D-GlcNAc present in outer core regions are the main MBL targets. Any steric obstacles within these motifs hinder MBL access, as was demonstrated for TS-OS1 core type of *H. alvei*. In TS1, the DS motif is substituted by terminal Gal residue that prevented hMBL binding to *H. alvei* PCM 1192 (Figure 5C).

Our results indicate a crucial role for MBL-binding motifs within the outer core OS in the recognition of *H. alvei* LPS by human and murine MBL, induction of an anaphylactoid reaction and rapid death in MDP-sensitized mice. Previously, it was demonstrated that such events were induced by several LPS of smooth bacteria with O-PS that were homopolymers of mannose (*K. pneumoniae* O3 and O5), able to activate complement *via* the LP (16). Among six *H. alvei* LPS with different O-serotypes tested, the intravenous injection of DS-containing LPS (PCM 1200, 1209, and 1190) led to development of severe symptoms of an anaphylactoid reaction and resulted in the death of animals within 30 min. Furthermore, *H. alvei* PCM 1212 (synthesizing Ra core type, with d-GlcNAc residue in the outer core region) is as toxic for mice as the aforementioned LPS. By contrast, *H. alvei* PCM 1192 LPS with outer core OS1 substituted with TS1 (preventing MBL binding) was not active. Interestingly, in the case of PCM 1200, 1212, and 1190 LPS, MBL-A was able to recognize not only the core OS (Ra or OS1-DS type) but also the O-PS region. Moreover, MBL-A showed the highest affinity to O-PS of *H. alvei* PCM 1200 LPS, which was found to be the most toxic (Table 2). Our results demonstrated that MBL-binding motifs in outer core region are sufficient to induce an anaphylactoid reaction in mice; however, the presence of S-LPS in the heterogeneous LPS preparation was mandatory. It might be suggested that similar to SP-D exhibiting O-PS-stabilized reactivity with common core OS of *K. pneumoniae, E. coli*, and *S. enterica* ser. Minnesota LPS, the O-PS–MBL interaction may also stabilize residual interactions of the collectin with the core OS region (54, 64).

The data presented here have extended the repertoire of LPS recognized by MBL, including rough forms present in endotoxin preparations from smooth bacteria (Figure 1). Generally, d-GlcNAc or Hep residues in the outer core were common ligands for the lectin. Those structures may be accessible to MBL *in vivo* not only when LPS O-PS is relatively short but also when endotoxin is released due to bacterial cell damage (for example, after treatment of host with antibiotics). We demonstrated also that the O-PS structure might augment immune responses when recognized by MBL (the example of PCM 1200). We believe that clarifying MBL specificity/affinity may contribute to a better understanding of the role of the LP in Gram-negative infections in general, including those leading to sepsis or endotoxic shock. Species of the family *Enterobacteriaceae* are responsible for 40–50% of hospital-acquired infections leading to sepsis and septic shock. Over half of cases in the USA is connected with bacteria of the genera *Klebsiella, Escherichia, Proteus* or *Enterobacter*, and mortality is in the range of 20–50%. In some cases of invasive infections caused, for example, by *K. pneumoniae, E. coli*, or *Proteus* spp., MBL/ficolin-dependent complement activation by common core oligosaccharide regions or MHP might contribute to the severity of infections and sepsis. Although interaction of MBL (or other PAMPs) with LPS is generally beneficial for the host, it may be harmful under certain conditions. Upon antibiotic treatment, aggregates of endotoxin (mixed S- and R-LPS) are released into the bloodstream and activate a host immune response (49). Furthermore, R-LPS was reported to exhibit higher potency in cell activation through the TLR-4/MD-2 receptor (65). Although MBL deficiency has been associated with susceptibility to infections (especially in children or immunocompromised subjects), its contribution to life-threatening events (like post-operative SIRS) has also been proven (66). Our results contribute to a better understanding of MBL–LPS interaction. They also support further development of therapeutic strategies against sepsis based on complement inhibition or complement-related replacement therapies.

## Ethics Statement

This study was carried out in accordance with the recommendations of Local Ethical Commission for Animal Experimentation with the headquarters in the Ludwik Hirszfeld Institute of Immunology and Experimental Therapy Polish Academy of Sciences (Wroclaw, Poland). The Local Ethical Commission for Animal Experimentation approved all *in vivo* protocols.

## Author Contributions

JL, AS, AM-K, MC, and CL conceived and planned the experiments. AM-K, AM, AS, and JL carried out the experiments with prevailing role of AM-K and AS. JL, MH, AM, and CL prepared OS1- or TS1-specific rabbit antisera. AM-K, AM, and JL isolated some *H. alvei* LPS. AR and MS isolated and provided the collection of *Proteus* spp. LPS. AM-K, JL, AS, and MC contributed to the interpretation of the results. JL, AS, MC, and AM-K took the lead in writing the manuscript. All the authors approved the manuscript and provided critical feedback.

## Conflict of Interest Statement

The authors declare that the research was conducted in the absence of any commercial or financial relationships that could be construed as a potential conflict of interest.

## Abbreviations

Ab, antibody; AP, alternative pathway; CP, classical pathway; DS, disaccharide; LP, lectin pathway; LPS, lipopolysaccharide, endotoxin; MBL, mannose-binding lectin; MASP, MBL-associated serine proteases; MHP, mannose homopolymer; NHS; normal human serum; OS, oligosaccharide; O-PS, O-specific polysaccharide; PAMP, pathogen-associated molecular pattern; R-LPS, rough LPS; S-LPS; smooth LPS; TS, trisaccharide.
