# Supplementary material for: Interaction of Mannose-Binding Lectin With Lipopolysaccharide Outer Core Region and Its Biological Consequences
Source: Front Immunol. 2018 Jun 29;9:1498. doi: 10.3389/fimmu.2018.01498 (PMC6033962; doi:10.3389/fimmu.2018.01498)
Supplement: Supplementary file 1 [file Table_1.pdf]

Supplementary Material

Interaction of Mannose-Binding Lectin with Lipopolysaccharide Outer Core Region and its Biological Consequences

Aleksandra Man-Kupisinska, Anna S. Swierzko, Anna Maciejewska, Monika Hoc, Antoni Rozalski, Malgorzata Siwinska, Czeslaw Lugowski, Maciej Cedzynski, Jolanta Lukasiewicz*

Correspondence: Jolanta Lukasiewicz: jolanta.lukasiewicz@iitd.pan.wroc.pl

Table S1. Serum hMBL interactions with core and O-PS regions of various LPS.

Interactions were detected with the use of SDS-PAGE/lectin blotting. Symbols +, ++, +++ stand for weak, moderate and strong reaction respectively. Abbreviations used: *K. p.*, *Klebsiella pneumoniae*, *P. m.*, *Proteus mirabilis*, *P. v.*, *Proteus vulgaris*, *P. p.*, *Proteus penneri*, *E. anguillimortifera*, *Edwardsiella anguillimortifera*, *E. coli*, *Escherichia coli*. Symbols in brackets represent serotype O.

bacteria	**core OS**	**O-PS**	**bacteria**	**core OS**	**O-PS**	**bacteria**	**core OS**	**O-PS**	**bacteria**	**core OS**	**O-PS**
K. p. 1 (O1)	+++		*P. m.* RMS 203 (O13)			*P. m.* 69/57 (O43)			*P. p.* 103 (O73 ab)	+	
K. p. 2 (O1)	+++		*P. v.* 30/57 (O15)	+++	++	*P. v.* 70/57 (O44)			*P. m.* CCUG 10705 [OF] (O74)	+	
K. p. 9 (ORa)	+++		*P. m.* 31/57 (O16)			*P. v.* CCUG 4680 (O45)			*P. m.* CCUG 10702 [OC] (O75)	+	
K. p. 11 (O3)	+++	+++	*P. m.* 61/57 (O17)			*P. v.* 71/57 (O47)			*P. v.* HSC 438 (O76)	+	
K. p. 13 (OR)			*P. m.* 34/57 (O18)			*P. m.* 75/57 (O49)			*P. m.* 3B-m (O77)		
K. p. 15 (O4)			*P. v.* 37/57 (O19a)			*P. m.* TG 332 [O203] (O50)			*P. m.* 1B-m (O78)	+++	
K. p. 27 (O2)	+++		*P. m.* 38/57 (O20)			*P. p.* 15 (O52)			*E. coli* 233		
K. p. 37 (O1)	+++		*P. v.* 39/57 (O21)	+		*P. v.* TG 276-1 (O53)			*E. coli* 399	++	
K. p. 43 (O2)	++		*P. v.* 40/57 (O22)			*P. m.* CCUG 10704 [OE] (O54 ab)			*E. coli* 820		
K. p. 57 (O5)	+++	++	*P. m.* 42/57 (O23 abd)			*P. v.* TG155 [O104] (O55)	++	++	*E. coli* 1238	+	
K. p. 58 (O3)	+++	++	*P. m.* 46/57 (O24)	+		*P. genomospecies* 4 (O56)			*E. coli* O39		
K. p. 61 (O5)	+++		*P. v.* 48/57 (O25)			*P. m.* TG 83 (O57)			*E. coli* O56	+	
K. p. 64 (O6 or O1)	+		*P. m.* 49/57 (O26)	+		*P. p.* 12 (O58)	+		*E. coli* R1		
K. p. 78 (O11)	++		*P. m.* 50/57 (O27)			*P. p.* 14 (O59)			*E. coli* R2	+++	
K. p. 80 (O12)	++		*P. m.* 51/57 (O28)			*P. myxofaciens* (O60)	+		*E. coli* R3	++	
P. v. CCUG 18984 (O1)	+		*P. m.* 2002 (O29 ab)			*P. p.* 21 (O61)		++	*E. coli* R4		
P. v. OX2 (O2)	++	+	*P. m.* 53/57 (O30)	+		*P. p.* 41 (O62)			*Citrobacter* Tcs		
P. m. 29906 (O3 ac)		+	*P. v.* 55/57 (O31 ab)			*P. p.* 22 (O63)			*Citrobacter* O16		
P. v. 9/57 (O4)	+	+	*P. v.* 57/57 (O32)	+		*P. p.* 40 (O64 abd)			*Citrobacter* 1560		
P. m. 13/57 (O5)			*P. m.* 59/57 (O33)			*P. p.* 34 (O65)			*Citrobacter* O32	++	
P. m. ATCC 49565 (O6)			*P. v.* CCUG 4669 (O34)			*P. p.* 2 (O66)			*Citrobacter* 114/66		
P. m. 16/57 (O7)			*P. v.* 72/57 (O37 ac)			*P. p.* 8 (O67)			*E. anguillimortifera* 1145	++	
P. v. 17/57 (O8)	+		*P. m.* 64/57 (O38)			*P. p.* 63 (O68)	+		*E. anguillimortifera* 1151	++	
P. m. 18/57 (O9)	+	+	*P. v.* 65/57 (O39)	++		*P. m.* TG 277 [O202] (O69)			*E. anguillimortifera* 1153	++	
P. m. HJ 4320 (O10)	+	+	*P. m.* CCUG 10703 [OD] (O40)			*P. p.* 60 (O70)	+		*E. anguillimortifera* 1158	+	
P. m. 24/57 (O11)		+	*P. m.* 67/57 (O41)			*P. p.* 42 (O71)	++				
P. v. 25/57 (O12)	+	+	*P. v.* CCUG 4677 (O42)			*P. p.* 1 (O72a)	+++				
